# Supplementary material for: COVID-19 Patients in the COVID-19 Recovery and Engagement (CORE) Clinics in the Bronx
Source: Diagnostics (Basel). 2022 Dec 30;13(1):119. doi: 10.3390/diagnostics13010119 (PMC9818274; doi:10.3390/diagnostics13010119)
Supplement: Supplementary file 1 [file diagnostics-13-00119-s001.zip › diagnostics-2045790-supplementary.pdf]

Supplementary Table S1. *Major lung imaging findings pre-, during and post-COVID-19 diagnosis broken down by hospitalization status. N reflects the number of unique patients. Post covid average 5 months after diagnosis (N=97).* * $p<0.05$, *** $p<0.001$

	**Pre-COVID-19**		**During-COVID-19**		**Post-COVID-19**	
	Hospitalized	Non-Hospitalized	Hospitalized	Non-Hospitalized	Hospitalized	Non-Hospitalized
Total N with imaging	48	16	70	5	57	13
No opacity	45	14	11 ***	4 ***	19 ***	11 ***
Opacity	3	1	59 ***	1 ***	38 ***	2 ***
Unilateral	1	1	7	1	9	0
Bilateral	2	0	52	0	29 *	2 *
No fibrosis	46 *	13 *	69	5	45	11
Fibrosis	2 *	3 *	1	0	12	2
